# A comparative Study of Novel Extramedullary Fixation and Dynamic Hip Screw in the Fixation of Intertrochanteric Fracture: A Finite-Element Analysis

**DOI:** 10.3389/fsurg.2022.911141

**Published:** 2022-05-25

**Authors:** Kai Ding, Yanbin Zhu, Haicheng Wang, Yonglong Li, Weijie Yang, Xiaodong Cheng, Yingze Zhang, Wei Chen, Qi Zhang

**Affiliations:** ^1^ Trauma Emergency Center, The Third Hospital of Hebei Medical University, Shijiazhuang, China; ^2^ Key Laboratory of Biomechanics of Hebei Province, Orthopaedic Research Institute of Hebei Province, Shijiazhuang, China; ^3^ NHC Key Laboratory of Intelligent Orthopaedic Equipment, the third hospital of hebei medical university, Shijiazhuang, China; ^4^ Chinese Academy of Engineering, Bingjiaokou Hutong, Beijing, China

**Keywords:** triangular structure, intertrochanteric fracture, finite-element analysis, biomechanical performance, DHS, TSFP

## Abstract

**Background:**

Dynamic hip screw (DHS) is one of the most widely internal fixations for stabilizing intertrochanteric fracture, however, with a high risk of postoperative complications. The triangle support fixation plate (TSFP) is developed to reduce the postoperative complications. The purpose of study is to evaluate the biomechanical performance of the DHS and TSFP and demonstrate the rationality of triangular internal fixation for stabilizing intertrochanteric fractures.

**Methods:**

The CT data of the proximal femur were used to establish finite-element models. Evans type I and IV intertrochanteric fracture were constructed and stabilized with the DHS and TSFP. The Von-Mises stress, maximum principal stress, minimum principal stress, and displacement were used to evaluate the biomechanical effect of two implants on intertrochanteric fracture.

**Results:**

Under a 600N axial load, the maximum stress and displacement of an intact proximal femur were 13.78 MPa and 1.33 mm, respectively. The peak stresses of the bone in the TSFP were 35.41 MPa and 68.97 MPa for treating Evans type I and IV intertrochanteric fractures, respectively, which were lower than those in the DHS. The maximum overall displacement and relative distance of the fracture surface in the DHS fixation model were 1.66 mm and 0.10 mm for treating Evans type I intertrochanteric fracture, which was 29.59% and 150% higher than that in the TSFP, and were 2.24 mm and 0.75 mm for treating Evans type IV intertrochanteric fracture, which was 42.58% and 650% higher than that in the TSFP.

**Conclusions:**

In conclusion, the TSFP has obvious advantages in stress distribution and stability than the DHS, providing a promising option for the treatment of intertrochanteric fractures.

## Introduction

As an osteoporosis-directly-related fracture, intertrochanteric fracture is highly prevalent in the elderly, accounting for approximately 3.4% of all fractures, and is expected to reach 4.6 million worldwide by the year 2050 (1, 2). Compared with the other type of hip fracture, femoral neck fracture, intertrochanteric fractures are associated with a higher risk of postoperative complications including mortality, with the latter reported at rates ranging from 11% to 29% within one year (3–6). Early surgical treatment within 24–48 h after injury is recommended as the standard intervention method to achieve quick functional recovery and reduced postoperative complications (7).

Among various operative methods, open reduction with DHS fixation is considered the gold standard for the treatment of stable intertrochanteric fractures (8–10). From the point of view of biomechanics, the DHS and other techniques reconstruct the anatomical structure of the tension trabeculae, and the fulcrum shifts outward, increasing the length of the force arm. The above internal fixation does not reconstruct the fracture of the tension line of the proximal femur (11, 12). Even if a slight load is applied to the femoral head, it can be amplified at the nail and plate, which can easily cause backout and hip inversion. Due to the limitations of the DHS per se in design, the DHS is related to various postoperative complications, including withdrawal, cut-out, and varus collapse, with fixation-failure rates of 1.5%–21%. It is suggested that posteromedial cortical integrity is critical to the clinical prognosis for the DHS fixing intertrochanteric fractures (13–16). In addition, lateral cortical integrity contributes greatly to stability, and the loss of lateral cortical integrity is reported to be associated with up to a 6-time risk of reoperation (10, 17).

Analyses of these failure cases indicated that the single fixation screw of the DHS could not support adequate force to counteract the fractured trabeculae, leading to direct hardware-related complications, and the ideal internal fixation devices should first be able to address this problem.

In this study, we will present our innovation, the TSFP, where the supporting screw is added to form a stable triangular structure with the main plate and fixing screw, thus enhancing the ability to resist the tension and compression force at the proximal femur (18–21). In 2009, our research team first proposed the concept of triangular support fixation that the anti-tension screw was added to form a double triangular structure with the main plate and anticompression screw (18, 19), and 3 Chinese granted patents were obtained (ZL200920254063.4, ZL200920254062.x, ZL201120370391.8). Theatrically, this will reduce the dependence on posteromedial and lateral cortical integrity and further minimize the occurrence of hardware-related complications.

In this study, our main aim was to compare the stress distribution and stability of two implant fixation models by using finite-element analysis and verify whether the innovated triangular support fixation could have superior biomechanics of intertrochanteric fractures compared with the traditional DHS. Finite-Element Analysis (FEA) is a numerical analysis method to simulate the real physical system (geometry and load conditions) by using mathematical approximation. It is widely used in the trauma field because of its advantages of simple operation, convenient model acquisition, and strong experimental reliability (11, 12, 22).

## Methods and Material

The study protocol was approved by the Institutional Review Board (IRB) and conformed to the provisions of the Declaration of Helsinki. Written informed consent was obtained from the volunteers prior to the study commencement.

### Three-Dimensional Models

A healthy volunteer (male, age 27 years, height 173 cm, and body weight 60 kg) without a history of lower extremity injury was included. The volunteer underwent a 64 slice spiral CT scan (SOMATOM Definition AS Siemens, Germany) with a slice of 0.625 mm from the hip joint to the knee joint. The CT data were used to establish a three-dimensional model of the proximal femur in Mimics 21.0 (Materialise company, Leuven,). The proximal femoral model was imported into Geomagic 13.0 (Geomagic company, USA) to generate the solid models. Hypermesh 2014 (Altair Company, USA) was used to mesh the solid model with C3D4 elements.

**Figure 1:**
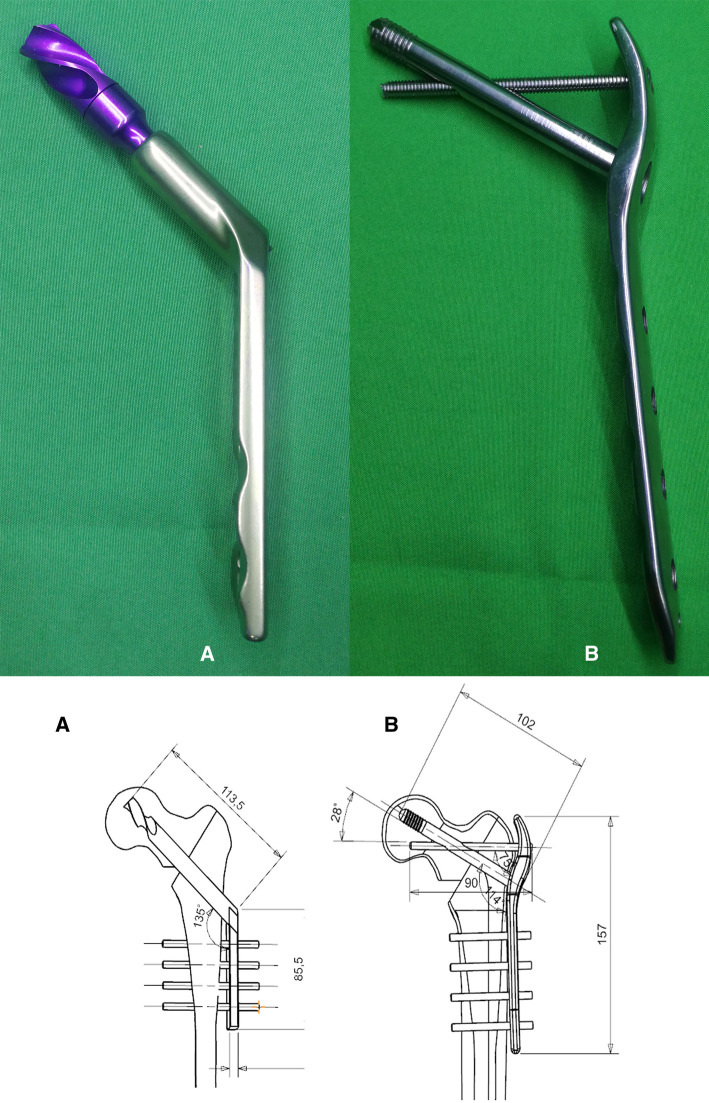
Diagram, detail, and specifications of internal fixation devices; (**A**) DHS; and (**B**) TSFP.

**Figure 2:**
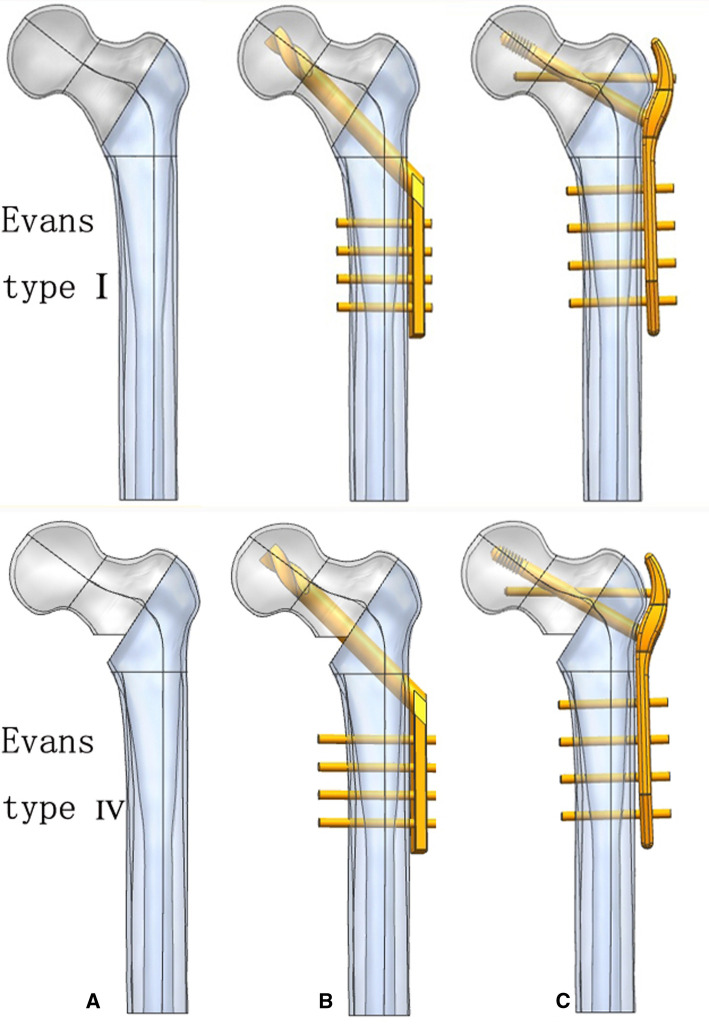
Geometric modeling of stable and unstable intertrochanteric fracture models and three implant fixation models; (**A**) the Evans type I and IV intertrochanteric fractures; (**B**) DHS fixation model; and (**C**) TSFP fixation model.

According to their real dimensions, the DHS and TSFP were constructed by UG-NX 12.0 (Siemens Company, USA). The length, dimension, and angle of the DHS and TSFP are demonstrated in Figure 1 (Figures 1, 2). We considered Evans I and IV intertrochanteric fractures as typically stable and unstable intertrochanteric fractures, respectively. To brief the research plan of this study, Evans type I and IV intertrochanteric fractures were included. These fractures were created and stabilized with the DHS and TSFP in UG-NX 12.0, respectively. The key point of the TSFP was an added supporting screw, which constituted a cross-structure with the locking screw through the fitting hole (sliding hole or threaded hole), forming a double triangle fixation of the main plate, locking screw, and supporting screw in a three-dimensional space.

### Material Properties

The models were imported into Abaqus6.14(Dassault Systèmes Solid Works Corp., Concord, MA, USA). The bone and implant were set as homogeneous, isotropic, and linear elastic materials. On the basis of previous literature, the Young’s modulus of the cortical and cancellous bone was 17 GPa and 1.5 GPa, while the Poisson’s ratio was 0.3 for both (23, 24). The Young’s modulus and Poisson’s ratio of the implant were 110 GPa and 0.316 (25), respectively.

### Boundary Conditions

**Figure 3:**
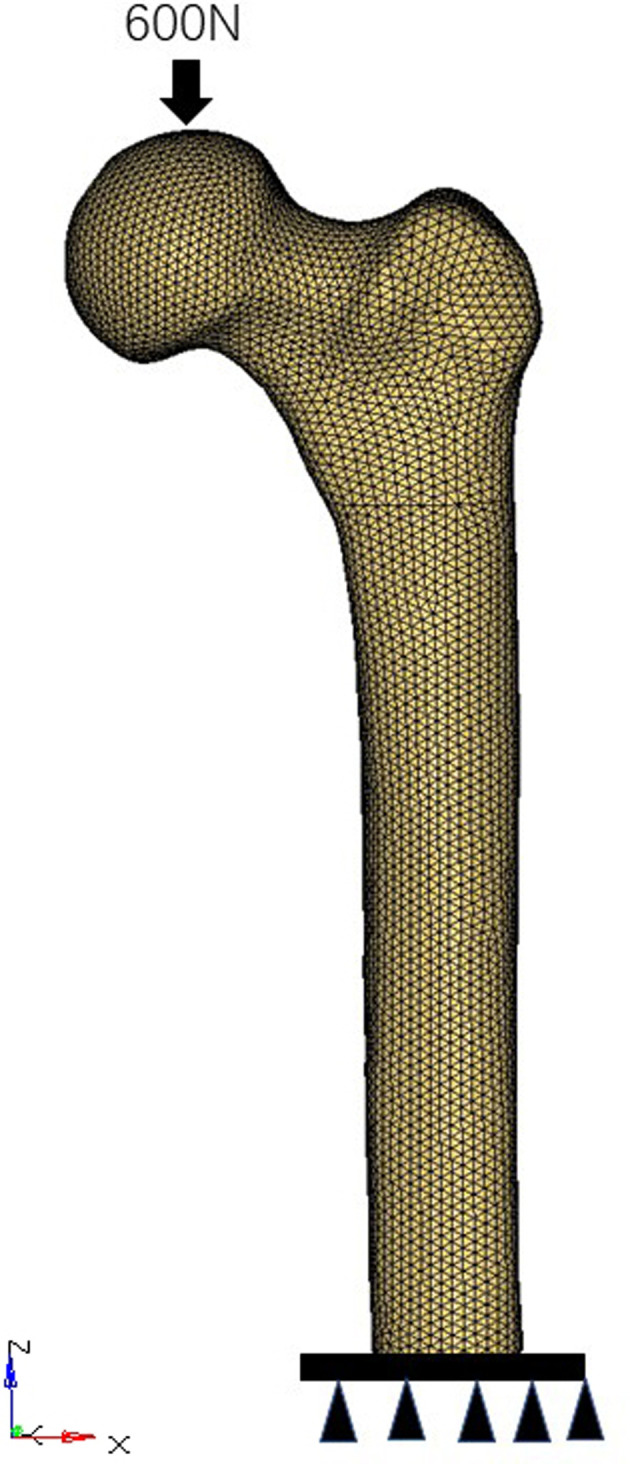
Boundary conditions of the models.

The full bonding was used as an interface between the supporting screw, fixating screw, and main plate in the TSFP model. The interfaces between the screw and the plate were assumed as fully bonding in the DHS model. The tied interactions between screw thread/bone and screw/ cortical bone were used to mimic the holding force. Other bone–screw and bone–bone interfaces were assumed as surface-to-surface contact relation. The friction coefficient was set as 0.3 (18, 26, 27). In this experiment, loads were applied using distributed coupling constraints. A concentrated force load was applied at the reference point, i.e., vertically above the femoral head. All construct models were applied with a load of 600N (one leg standing load force). The distal end of the femoral model was completely fixed in all degrees of freedom (Figure 3).

### Validation of Finite-Element Models

**Figure 4:**
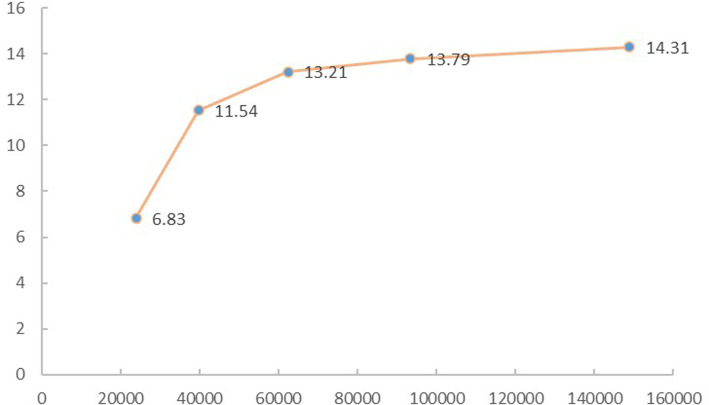
The von Mises stress on the cancellous and cortical bone of the proximal femur was tested to analyze the mesh convergence and validate the model.

In this study, we tested the maximum von-Mises stress on the cancellous and cortical bones of the proximal femur to analyze mesh convergence and validate the model. The selected method was widely used in the convergence test (27, 28). The convergence criterion used was a change of <5%. The final model was made of 7,564 nodes and 93,332 elements (mesh size: 2 mm) (Figure 4).

**Figure 5:**
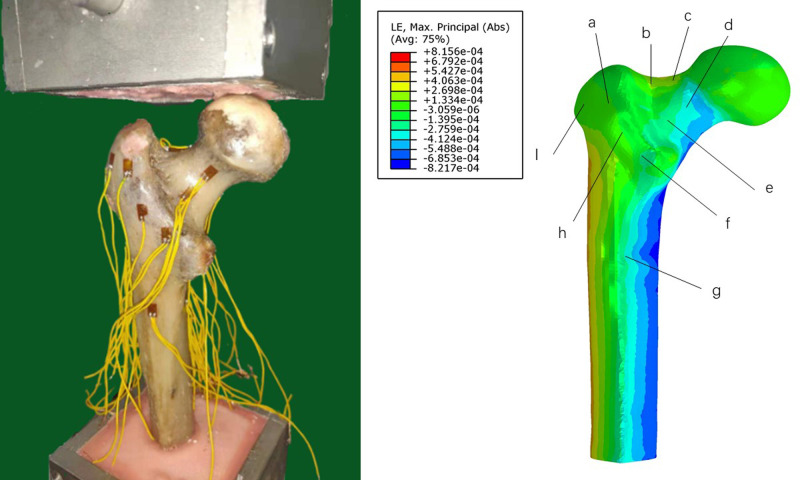
Results of the validated experiment. (**A**): biomechanical study; and (**B**): finite-analysis element.

**Figure 6:**
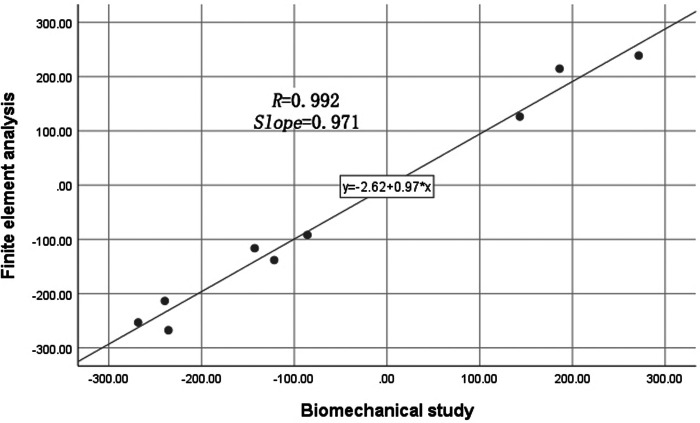
Recording the strain value of marker points in the biomechanical study and finite-element analysis.

The finite-element model was validated with a biomechanical study where the proximal femoral bone was fixed under the same boundary conditions. Femur specimens were selected from donated male cadavers. Specimens with rheumatism, tuberculosis, tumors, or abnormal bone quality were excluded using imaging examinations. The biomechanical testing machine (BOSE ElectroForce 3520-AT, BOSE Company, USA) was used to apply load. A 16-channel stress–strain tester (m + p, Hannover, Germany) and strain gauges (BF3503AA, Chengdu, China) were used to detect and record the stains of marker points. The strain values of nine marked points in the specimen were collected to compare with those in the finite-element analysis. From the comparison of the results, we found that our finite-element model of proximal femur was reliable and effective (Figures 5, 6 and Table 1).

**Table 1:** The strain values of the biomechanical test and finite-element analysis (10^−6^).

Maker point	a	b	c	d	e	f	g	h	i
Finite-element analysis	126.32	238.87	214.65	−253.24	−116.37	−267.54	−213.54	−91.94	−138.25
Biomechanical study	143.26	271.46	186.15	−268.39	−142.74	−235.74	−239.62	−85.83	−121.62

## Results

### The Von Mises Stress and Displacement Distribution of the Intact Bone

**Figure 7:**
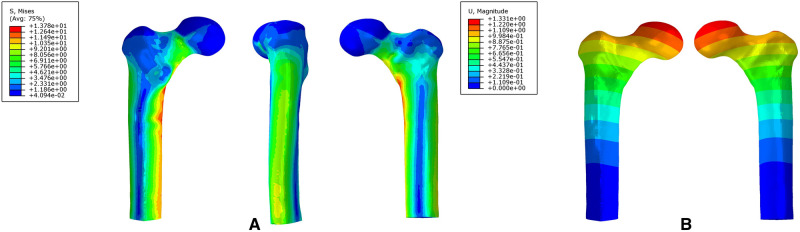
The stress distribution (**A**) and displacement distribution (**B**) of an intact proximal femur.

The maximum stress of the intact bone was 13.78 MPa, which was located at the medial femoral cortex. The maximum stress of the lateral femoral cortex was 6.31 MPa. The maximum displacement was 1.33 mm (Figure 7).

### The Von Mises Stress, Maximum Principal Stress, and Minimum Principal Stress Distribution of the DHS and TSFP Models for the Fixation of Evans Type I and IV Intertrochanteric Fracture

For the DHS and TSFP groups, a stress concentration of the medial and lateral femoral cortex occurred around the fracture and the hole adjacent to the main plate, respectively. The Von Mises stress, maximum principal stress, and minimum principal stress concentration of the DHS were located at the junction of the fixation screw and plate and those of the TSFP were placed on the junction of the supporting screw, fixating screw, and main plate.

**Figure 8:**
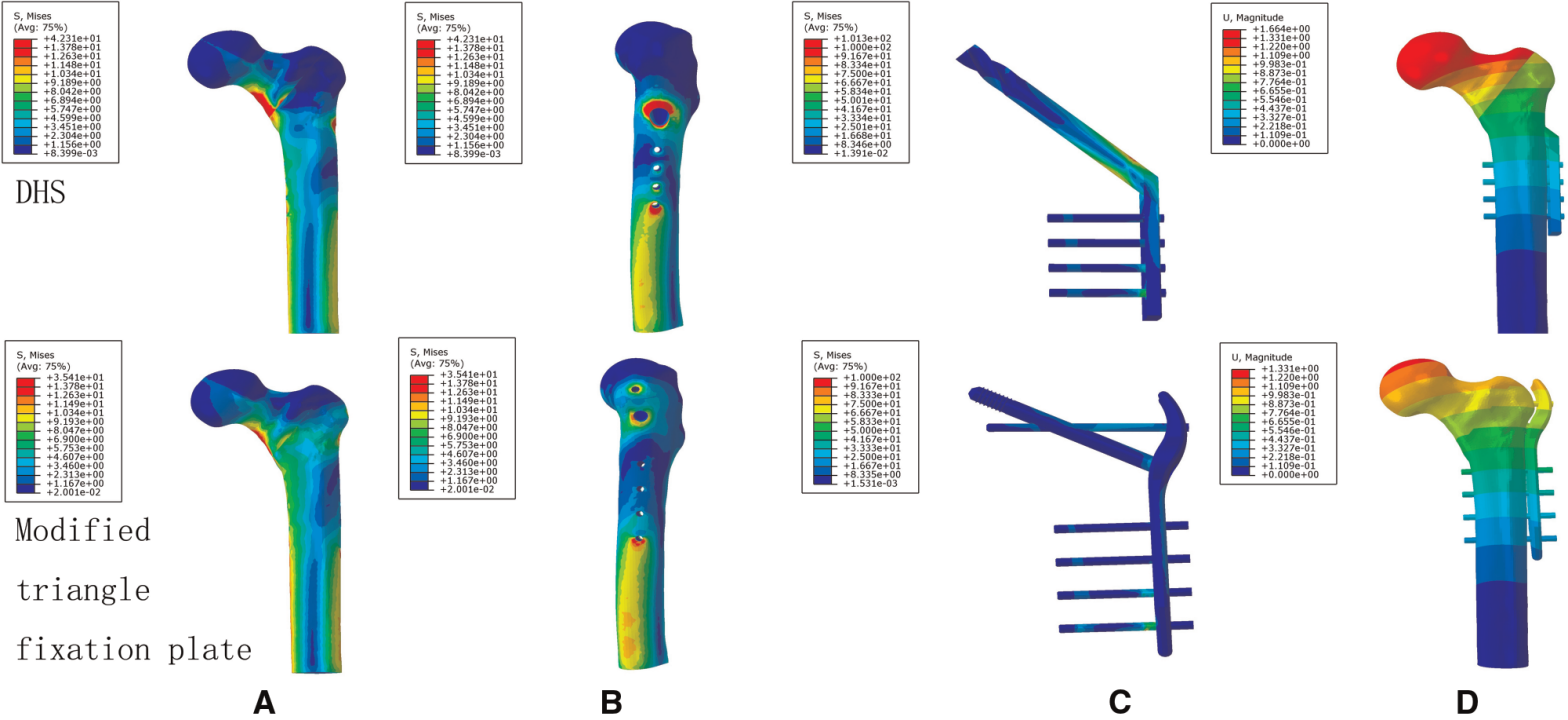
The stress distribution and displacement distribution of two implant fixations of a stable intertrochanteric fracture; (**A**) the stress distribution of bone models; (**B**) the stress distribution of the lateral femoral cortex; (**C**) the stress distribution of an implant model; (**D**) the displacement distribution of three implant fixation models. The figure shows that the TSFP has improved the stress distribution of the implant and bone models and increased the construct stability of the fixation of Evans type I intertrochanteric fracture.

**Figure 9:**
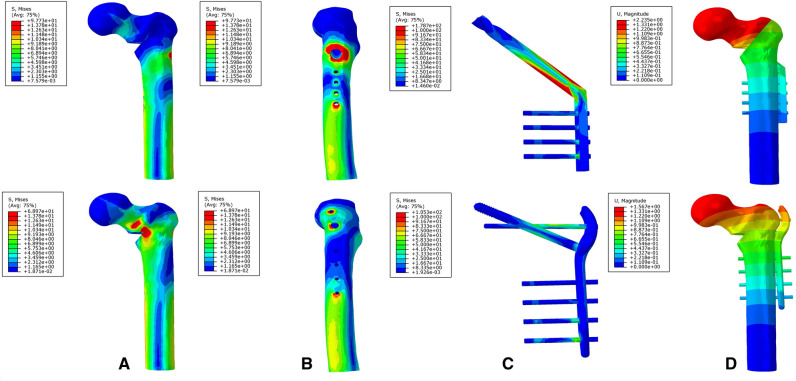
The stress distribution and displacement distribution of two implant fixations of an unstable intertrochanteric fracture; (**A**) the stress distribution of bone models; (**B**) the stress distribution of the lateral femoral cortex; (**C**) the stress distribution of an implant model; (**D**) the displacement distribution of three implant fixation models. The figure shows that the TSFP has improved the stress distribution of the implant and bone models and increased the construct stability of the fixation of Evans type IV intertrochanteric fracture.

**Figure 10:**
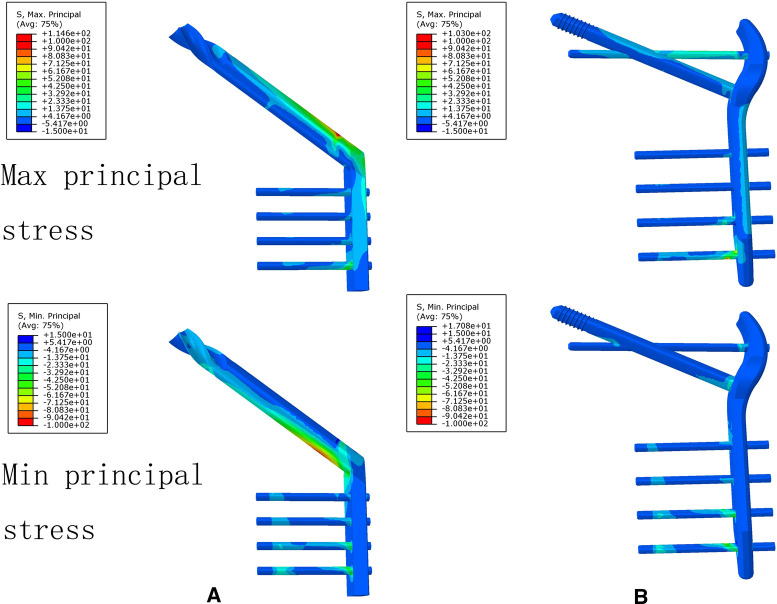
The maximum principal stress and minimum principal stress extremes of two implant models for the treatment of a stable intertrochanteric fracture. The figure shows that the TSFP has improved the maximum principal stress distribution compared with DHS for the fixation of Evans type I intertrochanteric fracture.

**Figure 11:**
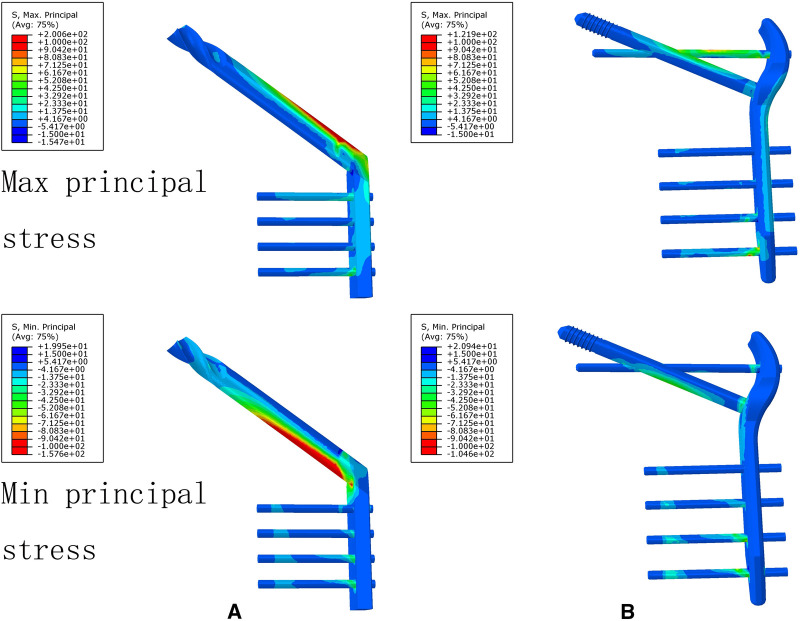
The maximum principal stress and minimum principal stress extremes of two implant models for the treatment of an unstable intertrochanteric fracture. The figure shows that the TSFP has improved the maximum principal stress distribution compared with DHS for the fixation of Evans type IV intertrochanteric fracture.

For Evans type I intertrochanteric fracture, the maximum mises stresses of the medial cortex, lateral cortex, and implant in the DHS were 1.19 times, 2.55 times, and 1.15 times greater than those in the TSFP, respectively. The DHS was 1.08 times and 1.11 times as much minimum and maximum principal stress extreme as the TSFP (Figures 8–11 and Tables 2–4).

For Evans type IV intertrochanteric fracture, the maximum mises stresses of the medial cortex, lateral cortex, and implant in the DHS were 41.66%, 4.99 times, and 1.70 times as much as that in the TSFP, respectively. The DHS was 1.51 times and 1.65 times as much minimum and maximum principal stress extreme as the TSFP (Figures 8–11 and Tables 2–4).

**Table 2:** The maximum displacement and stress values of two implant models for the treatment of stable intertrochanteric fracture.

Implant models	Maximum stress (MPa)			Maximum displacement (mm)	
	Medial cortex	Lateral cortex	Implant	Fixation models	Relative fracture surface
DHS	42.31	39.14	101.28	1.66	0.10
TSFP	35.41	15.36	88.02	1.28	0.04

**Table 4:** The maximum principal stress and minimum principal stress distribution of two implant models for the treatment of unstable and stable intertrochanteric fractures (MPa).

Implant models	Stable intertrochanteric fracture		Unstable intertrochanteric fracture	
	Max principal stress	Min principal stress	Max principal stress	Min principal stress
DHS	114.61	−89.65	200.63	−157.63
TSFP	103.04	−83.28	121.92	−104.55

### The Displacement Distribution of Two Implants for Treatment of Evans Type I and IV Intertrochanteric Fracture

For two variations, the maximum displacement values of the DHS for stabilizing Evans type I and IV intertrochanteric fractures were 1.66 mm and 2.24 mm and those of the TSFP were 1.28 mm and 1.57 mm, respectively.

The maximum relative displacements of the fracture surface of the DHS for fixing Evans type I and IV intertrochanteric fractures were 0.10 mm and 0.75 mm, respectively, and those of the TSFP were 0.04 mm and 0.10 mm, respectively (Figures 8, 9 and Tables 2, 3).

**Table 3:** The maximum displacement and stress values of two implant models for the treatment of an unstable intertrochanteric fracture.

Implant models	Maximum stress (MPa)			Maximum displacement (mm)	
	Medial cortex	Lateral cortex	Implant	Fixation models	Relative fracture surface
DHS	28.73	97.73	178.74	2.24	0.75
TSFP	68.97	19.58	105.31	1.57	0.10

## Discussion

In the current study, we found that the TSFP showed superior biomechanical performances, including reducing the reliance of the DHS on the posteromedial and lateral femoral cortex, increasing the construct stability, and improving the stress distribution of the implant. The main reason for the improved biomechanics of the implant construct model is the cross structure of the supporting and fixating screws. The concept of triangular support fixation would be important to improve clinical outcomes and reduce postoperative complications in treating various intertrochanteric fractures.

The TSFP provides a better biomechanical performance than the DHS in terms of mechanical aspects. This is due to the unique double triangular structure of the TSFP. First, the first triangle is composed of the cancellous bone of the femoral head, supporting screw, and fixing screw, which is called a mixed triangle. The second triangle, called a metal triangle, is constructed by simulating the triangular cantilever structure of the proximal femur which is made up of the supporting screw, fixing screw, and main plate. The fixing and supporting screws are kept in line with the spatial position and orientation of the trabecular system of the proximal femur. According to our result, the TSFP has less relative distance of fracture section, which shows that the mixed triangular structure can obviously improve the holding force of the fracture fragment. Secondly, the metal triangle plays the similar role of the screw-plate joint of the DHS, and three fulcrums of the metal triangle greatly disperse the stress concentration of the screw-plate joint. Moreover, the supporting screw and main plate together support the fixing screw, forming a double-pivot fixation to improve the overall stability of the intertrochanteric fracture. The triangle structure of the TSFP has better resistance to the tension and compression force in the proximal femur than the single fixing screw, reducing tension and compression force component to the femur which has lower requirement on the integrity of femoral posteromedial and lateral cortex. The double triangular structure perfectly solves the stress concentration of the screw-plate junction, reduces excessive dependence on the posteromedial and lateral cortex, and increases the stability of the fracture fixation model compared with the DHS, which has important clinical implications for reducing the risk of postoperative reduction loss, varus collapse, screw cut-out, and so on.

The biomechanical difference between the DHS and the TSFP was a result of varying the fixation mechanism for intertrochanteric fracture. The fixation mechanism of the DHS for intertrochanteric fracture was summarized as “two points and one line.” That is, one line is the fixing screw, and the two points are the posteromedial and lateral cortex of the proximal femur. Due to a huge bending moment of the proximal femur, body loading was transmitted to the tension and compression force, which single fixation screw could not fully counteract. Also, the posteromedial and lateral cortex in the proximal femur act as fulcrums to counteract the tension and compression force component, respectively. Biomechanically, only if the support of both posteromedial and lateral femoral cortex is maintained, the DHS fixation of intertrochanteric fractures will be safe (29). Therefore, the peak stresses of the posteromedial and lateral femoral cortex in the DHS fixation model were 42% and 4.99 times of those in the TSFP for treating an unstable intertrochanteric fracture, which could be explained by the fact that the DHS construct model had a higher instability and separation of the fracture fragment than that of the TSFP with the loss of the posteromedial femoral cortex. The triangle structure is more suitable for intertrochanteric fracture than for a single fixing screw.

The literature review showed that clinical effects were increasing with the continuous development of extramedullary devices from the DHS to the MSP (Medoff sliding plate) and the LCP to the PCCP (percutaneous compression plating system) (30). However, the above instrument was not currently used for the main treatment of intertrochanteric fracture. Although the MSP and LCP increased the construct stability and reduced the risk of fixation-related complications, intraoperative soft tissue dissection and bleeding were difficult to avoid because of the complex surgical procedure (31–33). The PCCP achieved a minimally invasive procedure, but its double parallel lag screw still could not solve the long lever arm and stress concentration of the screw-plate junction (34, 35). Intramedullary fixation has gradually become the mainstream treatment for intertrochanteric fractures due to minimal invasion, small soft tissue injury, and shortening of the long arm (36, 37). However, the single fixing screw of Gamma nail and PFNA was also not counteracted with the tension and compression force of the proximal femur like the DHS, leading to an overreliance on the femoral cortex, whose fixation-related failures reached between 2.5% and 12.5% (30, 38–40). Although there was an in-depth understanding of the trabecular system of proximal femur, the concept of triangular support fixation was first put forward by our research team, and 3 Chinese granted patents were obtained in 2009. The TSFP had high stability, combining the advantages of intramedullary and extramedullary fixation. We considered that the TSFP was a bionic internal fixation of the proximal femur, which could be suitable for various intertrochanteric fractures, and had a great impact on the prognosis of intertrochanteric fractures. Finally, we will also carry out the biomechanical and clinical studies of the TSFP and promote the clinical application of the TSFP in intertrochanteric fracture.

There are some limitations in this study. First, the properties of the bone were set as homogeneous, isotropic, and linear elastic behavior, which were different from those of the actual bone. However, the bone model was validated with the biomechanical study, and the difference was within the acceptable range (*R *= 0.992, *Slope *= 0.971). Second, we explored the optimal biomechanical effects of the different implantation points of the supporting screw versus the fixation screw in the lateral wall on intertrochanteric fractures. Third, because different intertrochanteric fractures have its own unique characteristics, the absence of an Evans II/III/V intertrochanteric fractures model in this study limited the value of our research. Finally, the four-hole side-plate DHS selected in the study was one fixation type in clinical practice, but to the question whether the DHS was the optimal fixation for intertrochanteric fractures, there was a lack of biomechanical and clinical evidence (41–43). In addition, the DHS has a variety of structures and material properties, which may impact the results of this study.

In conclusion, the TSFP reduced dependence on the femoral posteromedial and lateral cortex and improved the stability and stress distribution of the construct model. The TSFP conformed to the biomechanical characteristics of the proximal femur, which is a promising internal fixation for intertrochanteric fractures.

## Data Availability

The original contributions presented in the study are included in the article/Supplementary Material; further inquiries can be directed to the corresponding author/s.
